# Frontline Health Service Providers’ Perspectives on HIV Vaccine Trials among Female Sex Workers and Men Who Have Sex with Men in Karnataka, South India

**DOI:** 10.1371/journal.pone.0141822

**Published:** 2015-10-30

**Authors:** Satyanarayana Ramanaik, Leigh M. McClarty, Shamshad Khan, B. M. Ramesh, Monika Doshi, Marissa L. Becker, Robert R. Lorway

**Affiliations:** 1 Centre for Multi-Disciplinary Development Research, Dharwad, Karnataka, India; 2 Karnataka Health Promotion Trust, Bangalore, Karnataka, India; 3 Centre for Global Public Health, Department of Community Health Sciences, University of Manitoba, Winnipeg, Manitoba, Canada; 4 Department of Communication, University of Texas at San Antonio, San Antonio, Texas, United States of America; 5 Department of Medical Microbiology, University of Manitoba, Winnipeg, Manitoba, Canada; University of Toronto, CANADA

## Abstract

**Background:**

Little qualitative research is available on the role of frontline health service providers (FHSPs) in the implementation of clinical trials, particularly in developing countries. This paper presents findings from a qualitative study about the perspectives of FHSPs on future HIV vaccine trials involving female sex workers (FSWs) and men who have sex with men (MSM) in three districts of Karnataka, India. In particular, we explore FHSPs’ knowledge of and views on clinical trials in general, and examine their potential willingness to play a role if such trials were introduced or implemented in the region.

**Methods:**

A field team of four researchers from Karnataka—two of whom self-identified with FSW or MSM communities (“community researchers”) and two with backgrounds in social work—conducted in-depth interviews with FHSPs. Including community researchers in the study helped to build rapport with FSW and MSM participants and facilitate in-depth discussions. A coding scheme for transcribed and translated data was developed using a framework analysis approach. Data was then analysed thematically using a combination of a priori and emergent codes.

**Results:**

Over half of FHSPs demonstrated limited knowledge or understanding of clinical trials. Despite reported skepticism around the testing of HIV vaccines in developing countries and concerns around potential side effects, most FHSPs strongly advocated for the implementation of HIV vaccine clinical trials in Karnataka. Further, most FHSPs expressed their willingness to be involved in future HIV vaccine clinical trials in varying capacities.

**Conclusion:**

Given that FHSPs are often directly involved in the promotion of health and well-being of FSWs and MSM, they are well-positioned to play leadership, ethical, and communicative roles in future HIV vaccine trials. However, our findings reveal a lack of awareness of clinical trials among FHSP participants, suggesting an important area for capacity building and staff development before viable and ethical clinical trials can be set up in the region.

## Introduction

Vaccines can be extremely effective in preventing infectious diseases in a wide variety of geographic, economic, and societal contexts [1]. With respect to HIV, studies from the International AIDS Vaccination Initiative (IAVI) suggest that a vaccine with 60% efficacy, when effectively implemented, could reduce new HIV infections by 25% in its first decade and by almost half in 25 years [2,3]. In India, which ranks as having the third highest estimate of people living with HIV in the world [4], the development and successful implementation of an HIV vaccine could have a major impact on national HIV prevention efforts [5–7]. Furthermore, because the HIV epidemic in India disproportionately affects female sex workers (FSWs) and men who have sex with men (MSM) [8], these communities could particularly benefit from the development of an effective HIV vaccine.

In early 2006, the first HIV vaccine trial in India began [9] and since then, three additional phase I vaccine trials have been conducted in the country [6,10–12]. A private-public partnership between IAVI, the Indian government, and non-governmental partners has been working to create a platform to conduct large-scale vaccine trials in India [7,13]. However, India has been plagued by a history of unethical clinical trials [14–20]. In 2001, an experimental anti-cancer drug developed at Johns Hopkins University was tested by the leading Indian cancer institute, the Regional Cancer Centre (RCC) in Kerala [21]. The trial was halted amid accusations that the Government of India had not obtained proper informed consent, that surgery or other potentially life-saving conventional treatments had been delayed because of the administration of the experimental drug, and that the drug had not been properly screened for toxicity [22,23]. More recently, in early 2010, the Indian government documented misconduct due to the death of four young girls enrolled in a clinical trial for the human papillomavirus (HPV) vaccine in Andhra Pradesh and Gujarat [24]. Further enquiries revealed that study participants did not fully comprehend the risks of vaccination, despite signing a consent form, and ultimately, the study was halted due to ethical breaches [24,25]. While the deaths of the four participants were not attributable to the vaccine being tested, misconceptions brought about by media coverage of the event lead to the conflation of the two events by the general public.

Changes in the regulation of clinical trials in India over the last few years, although characterized by some commentators as overly restrictive, not only ensure greater transparency through mandatory registration, but the Indian Council of Medical Research (ICRM) now explicitly holds scientists running trials more accountable to the protection of participants’ rights and safety [26–28]. To begin to consider how future HIV vaccine trials and immunization programs in India can incorporate critical ethical issues, our study sought to explore the perceptions of frontline health service providers (FHSPs), who have worked over an extended period of time with marginalized communities of FSWs and MSM in the southern Indian state of Karnataka. In this region, there is a sizeable pool of FHSPs, including community-level peer leaders and outreach workers (ORWs), who have been working closely with FSWs and MSM over a ten-year period on the India AIDS Initiative known as *Avahan* [29]. Given their rich experience and long-term rapport with FSWs and MSM, we reasoned that FHSPs could provide important insights into processes involved in conducting clinical trials and would serve as an important resource for conducting HIV vaccine trials in India—both for improving participation and retention, while ensuring ethical implementation [30]. Previously conducted clinical trials have highlighted the involvement of FHSPs to maximise the enrolment and retention of trial participants and to ensure the rapid translation of research findings into acceptable programs [31].

In this paper we present findings from our qualitative study that sought to understand the perspectives of FHSPs on future HIV vaccine trials with FSWs and MSM in three geographically distinct districts of Karnataka. Specifically, we explored FHSPs’ knowledge about and views on clinical trials and aimed to better understand their potential willingness to play a role in the development and implementation of future HIV vaccine clinical trials. Our findings not only offer insights into how FHSPs may contribute to future HIV vaccine trials in Karnataka, but also into how they may also be able to inform strategies that ensure the ethical implementation and delivery of a future HIV vaccine.

## Methods

### Ethics statement

Ethical approval for this study was obtained from the University of Manitoba’s Health Research Ethics Board in Winnipeg, Canada, and from the Institutional Review Board at St. John’s Medical College in Bangalore, India. Written informed consent was obtained from all participants in their preferred language–either *Kannada* (Karnataka’s local language) or English.

### Study design and sampling methodology

This qualitative, exploratory study was designed with open-ended questions that aimed to gain an in-depth understanding of the perspectives of FHSPs with respect to hypothetical, future HIV vaccine trials conducted among FSWs and MSM. Specifically, FHSPs were asked about their knowledge of how new medicines or other biomedical technologies are tested for safety and effectiveness, their opinions about conducting clinical trials that target FSWs and MSM in Karnataka, and their willingness to be involved in future HIV vaccine trials—either as participants or in an administrative capacity. This study was part of a larger, multi-country ethnographic research project that sought to understand the extent to which FSWs and MSM are prepared for hypothetical HIV vaccines trials and vaccination programs. This phase of research was preceded by participant observation, which generated field notes upon which in-depth interview questions, and their wording, were based.

A total of 50 FHSPs were interviewed from three districts in Karnataka–Bellary (*n* = 15), Belgaum (*n* = 15), and Bangalore (*n* = 20). We defined a FHSP as anyone working in an organisation affiliated with the Karnataka Health Promotion Trust (KHPT)—an international non-governmental organisation acting as a state-level implementing partner for the *Avahan* programme [32]—or Karnataka State AIDS Prevention Society (KSAPS)—the state-level government body responsible for implementing India’s national HIV prevention and treatment programming [33]—who is in regular, direct contact with FSWs and/or MSM for the provision of HIV prevention and care services, or, anyone who makes decisions that directly impact the lives of FSWs and MSM that utilise HIV-related services in their organisation. Six categories of FHSPs were included in this study: (1) local program managers, (2) peer educators, (3) outreach workers, (4) counsellors, (5) doctors, and (6) nurses. It should be noted that many FHSP participants also self-identified as FSWs or MSM.

We employed purposive sampling to recruit FHSPs who had been working with communities of FSW and/or MSM for a minimum of one year. All FHSPs were at least 18 years old, and were able to provide informed consent. Participants were offered a small gift, worth 250 INR (approximately 5 USD), as compensation for their time and travel.

### Research team and training

The research team consisted of four researchers from Bellary, Belgaum, or Bangalore; two of whom self-identified with FSW or MSM communities (“community researchers”), and two had backgrounds in social work. Including community members as interviewers helped us to build greater rapport and facilitated in-depth discussions with FSW and MSM participants. In lieu of a piloting process, mock interviews were conducted with the research field team as a way to refine and receive feedback on the wording, composition, and structure of interview guides. Input from the two community researchers was particularly valuable during this process as they were able to ensure that the tool would be accessible for study participants who also identified with FSW or MSM communities. All four interviewers also underwent intensive training on research ethics and qualitative research methodologies, including interviewing techniques.

### Data collection

Prior to data collection, formal approval was granted from the respective organisations at which FHSP participants worked. Data was collected from August through September 2012. In-depth interviews, which lasted approximately 45–60 minutes, were conducted following written informed consent and were conducted in participants’ preferred language—Kannada or English. All interviews were audio-recorded and conducted in places where participants felt most safe and comfortable to openly discuss their views.

### Data analysis

Interviews were transcribed verbatim and those conducted in Kannada were translated into English. All transcribed and translated interviews were reviewed by the first author, who also consulted with the interviewers for accuracy and completeness. Transcripts were then imported into NVivo9 [34] and explored using narrative thematic analysis [35]. A framework analysis approach [36] was employed, in which an a priori coding scheme and definitions were developed by the first and last authors based on participant observation field notes, the interview guide, and available literature. The coding scheme was refined as themes that emerged during analysis. Transcripts were coded independently by the first and last authors using the coding scheme and definitions. The minor discrepancies that arose between coders were resolved through discussions with the second author. While the diversity in the sample of FHSPs provided richness to our data, thematic saturation was observed during the analysis of the 50 transcripts.

## Findings

### Socio-demographic characteristics

A total of 50 FHSPs were interviewed for this study. Socio-demographic characteristics of participants are listed in Table 1.

**Table 1:** Sociodemographic characteristics of research participants in Bellary, Belgaum, and Bangalore, India (*n* = 50).

Characteristics		*n*	%
**Employment**	Program manager	13	26
	Peer educator	10	20
	Outreach worker (ORW)	10	20
	Counsellor	9	18
	Doctor	7	14
	Nurse	1	2
**Age (years)**	Mean (range)	33 (22–48)	
**Sex**	Male	20	40
	Female	30	60
**Education**	No formal schooling	1	2
	Primary (1–7)	7	14
	Secondary (8–10)	11	22
	Pre-University Course (PUC; 11–12)	5	10
	Post-secondary	26	52
**Years of work experience in current organization**	1	6	12
	2–4	17	34
	≥5	26	52
**Nature of work**	Work directly with FSWs only	28	56
	Work directly with MSM only	12	24
	Work directly with FSWs & MSM	8	16
	Only involved in decision-making that impacts FSWs/MSM	2	4
**Type of organization**	Community-based Organization (CBO)	38	76
	Non-governmental Organization (NGO)	6	12
	Government institution	6	12

### Awareness of clinical trials

More than half of study participants had little to no awareness about what a clinical trial generally entailed, or about the clinical trials that have been conducted in India in the past. To better understand FHSPs’ general understanding of clinical trials, they were asked, “What do you know about research that tests new medicines in human beings, specifically in FSWs/MSM?”. The wording of this question was based upon findings from field notes obtained through participant observation, during which time it became evident that most FHSPs were not familiar with the English term “clinical trial”, nor the Kannada translation. The following narratives were received in response to this question: “*I don’t know if it has come in India or not” (ORW*, *CBO*, *Bangalore)*; *“I don’t know very clearly about those things” (Peer educator*, *CBO*, *Bellary);* and “*I tried to know about such research but haven’t gotten* … *much information about these kinds of research*.*” (Doctor*, *CBO*, *Belgaum)*. These findings were consistent across the diverse educational backgrounds and FHSP roles.

Even among participants who were aware of clinical trials, many confused them with ordinary medical tests and procedures routinely encountered in clinical settings.

*Medical research* … *in our [HIV] program*, *in our STI clinic*, *we do speculum examination of the cervix for herpes*, *non-herpes*, *vaginal discharge*. *I mean we give tablets for such sexually transmitted infection*. *(Counsellor*, *CBO*, *Bellary)*

*Yes*. *There was a friend of mine who was suffering from cancer*. *They took a sample of blood and sent it to Bangalore for testing*. *Later*, *they had started medication*. *Within six or seven months she completely recovered*. *(Peer educator*, *CBO*, *Belgaum)*

In general, there was a lack of awareness around the scientific process that runs human clinical trials testing the efficacy of new medical substances.

Among those who had some knowledge of the existence clinical trials—which mostly included FHSPs with higher levels of formal education (i.e., post-secondary degrees)—many admitted that they possessed limited information.

Among the few participants who had some awareness of clinical trials research, they shared their knowledge of an HIV microbicide trial that was halted, due to poor health outcomes that ran in the neighbouring district:

… *In Bagalkot [northern Karnataka] a study on microbicides was taken up but it was a pilot project that wasn’t continued*. *It wasn’t sanctioned for our district* … *we have just heard about that*, *we don’t have a clear idea [of what happened]* … *(Program manager*, *NGO*, *Belgaum)*

*[R]esearch was conducted in Bagalkot district which was called the ‘Gel Project’*. *That research had aimed to replace condoms with a gel to prevent HIV*. *However*, *the society didn’t accept it as there were so many adverse side-effects*. *(Counsellor*, *Government institution*, *Belgaum)*

This limited awareness of clinical trials likely can be attributed to participants’ limited prior exposure to, or involvement in, clinical trials, as well as the limited provision of reliable information about them. Therefore, with respect to site preparedness for clinical trials research, the education of local FHSPs who are directly or indirectly involved with developing and/or implementing clinical trials will be paramount. Media sources, such as newspapers, local television programming, and the Internet, seem to play an important role in connecting FHSPs in Karnataka with information about modern medical technologies and in disseminating scientific knowledge to the general population. When participants were asked, “Where did you learn about vaccines?”, the following responses were received: *“I read about vaccine in the newspaper” (Project manager*, *CBO*, *Belgaum)* and *“I have seen [information about vaccines] on TV” (Counsellor*, *CBO*, *Bangalore)*.

Among the few participants who possessed some awareness around clinical trials, their understanding was based on a phase III clinical trial that tested the efficacy of a vaginal microbicide among women characterised as being at high risk for HIV [37]. Conducted in a neighbouring district in northern Karnataka, the trial was halted prematurely due to an observed increase in HIV infection in the treatment arm [37].

### Views on clinical trials and HIV vaccines

After the interviewer provided the participants with basic definitions around medical research and clinical trials that test new HIV vaccines, participants were asked for their opinions on this type of research. A few FHSPs maintained negative views. For example, one doctor expressed concerns about conducting clinical trials involving human subjects in “developing” countries:

*It has been heard that HIV vaccine research is going on* … *such research is concentred in Third World countries* … *they don’t accept it in Western countries*. *Here*, *there are uneducated people* … *they [foreign researchers] bring [vaccines] and do research* … *this is wrong*. *(Doctor*, *CBO*, *Bangalore)*

This quotation speaks to larger debates about the ethics of research in “developing” countries [38]. In India, this concern for exploitation by foreign research was reported among MSM in Chennai and Mumbai [6]. However, Chakrapani et al. [39] insist that India imposes reasonably strict regulatory mechanisms that require “technical and ethical clearance from the government, with approval by the Indian Council of Medical Research (ICMR)” (p. 739).

Among participants who held negative perspectives on clinical trials to test HIV vaccines, side-effects were the biggest concern. Interestingly, some of these concerns were linked with their experience of working with people taking antiretroviral therapies (ART) and their knowledge of opportunistic infections:

*I have a fear of side-effects* … *See*, *some members of our community are already on ART*, *we usually witness the side-effects with these ART medicines*. *Similarly*, *I have a doubt about [vaccine] research whether it may create the same problems*. *We have the intention of doing something good for people*, *[but] I suspect that these new medicines have side-effects or what* …. *It may lead to TB*, *jaundice*, *skin diseases*, *burning and itching sensation in the legs*, *headache*, *heart diseases and kidney problems*, *liver problems*. *Like this there could be some side-effects…*. *(ORW*, *CBO*, *Bellary)*

Some FHSPs expressed that they feared losing the trust of the community in the event of side-effects:

*Sometimes I feel that this can be harmful*, *because such research may or may not be successful*, *it may fail*. *So we may involve the public in large numbers*, *thinking that it will be good for them*. *But if there is an* adda parinama *[a Kannada phrase referring to adverse reactions to a drug] of this product*, *then [the participants] may complain against us*, *and also it can create problems for the researchers as well*. *(ORW*, *CBO*, *Bangalore)*

In a poignant remark, one program manager cautions against focusing clinical trials solely on FSWs, suggesting that this could re-stigmatize an already marginalized group.

*Research should be carried out in the society*, *but it should not consider that the women are the root cause for initiating the kind of research*. *For example* …, *the government had firstly highlighted HIV by stating that this infection has been found in a female sex worker in Chennai* …. *In Karnataka also*, *it has been found in a female sex worker* …. *The public decided that HIV always affects female sex workers without fail*. *The same message was evolving in the public* … *for a decade*. *When we tried to convince the public that HIV will not affect FSWs alone*, *they were not in a position to believe it* …. *[W]hile giving a message to the society we should be very careful*, *and focus should be on reducing the prevalence of HIV*. *(Program manager*, *NGO*, *Belgaum)*

Despite these concerns, most participants felt it was highly important to have such research conducted among FSW and MSM communities, due to the disproportionate burden of HIV among these groups. For instance, one ORW said:

*I am not very aware of how clinical trials are conducted*. *But from the community point of view*, *I feel it is important to have such research to find solutions for* … *issues like HIV/AIDS*. *(ORW*, *CBO*, *Belgaum)*

A counsellor felt that marginalized communities could greatly benefit if a new vaccine to prevent HIV was developed.

*There are good people in the community; [however]*, *sometimes their clients will not agree to use condoms* … *If such vaccine is made available to them*, *they will become tension-free and stay healthy*. *It is more helpful to such communities*. *(Counsellor*, *NGO*, *Bangalore)*

A few participants expressed concerns that an HIV vaccine may actually increase unprotected sexual practices and sexual activity more generally.

*And in another way it is very harmful because the youngsters think that they don't get excitement if they do sex by using the condoms and then they take advantage of availability of such vaccines and do unsafe sex more and more*. *(Counsellor*, *CBO*, *Bellary)*

It is important to note that in quantitative research linked to this qualitative study, sexual disinhibition was also reported by FHSPs as a barrier to their recommendation of a future HIV vaccine to FSWs and MSM [25].

When asked about their perspective on HIV vaccine trials, one doctor specifically emphasized that an HIV vaccine would be helpful because it could “protect” FHSPs working with FSWs and MSM:

*Female sex workers are getting [infected with HIV] because of their risk behaviour*. *But we as* … *innocent doctors are also at risk and it is because of someone else’s mistakes*. *For example*, *recently a woman came for the delivery [of her baby] and she came without any HIV testing report* …. *In those cases*, *as doctors*, *we have lots of chances to get HIV*. *So*, *it is very important to find a vaccination for HIV so that it will be useful for everyone*. *(Doctor*, *Government institution*, *Bellary)*

Although the concern around risk of infection among clinicians who work closely with FSWs and MSM did not emerge in other interviews, the extent to which FHSPs see themselves as potential beneficiaries of an HIV vaccine—and how this shapes their motivation to promote future clinical trials—warrants further exploration. Interestingly, previous research related to this study found that over eighty percent of FHSPs in Karnataka indicated that they would be very likely to accept an HIV vaccine for themselves if one were to become available [40].

In contrast, a number of participants expressed considerable sensitivity toward the struggles that FSWs and MSM encounter. For instance, one doctor expressed concern for the fears and anxieties of the community members that they serve.

*It will be good if a new medicine is found and released*, *because most of the patients who have seen about this on television and at other places keep asking us*, *‘Madam*, *is there any medicine available to cure the disease’*. *We tell them that the experiments are going on and they too feel that it will be good if a medicine is made available for them*. *(Doctor*, *Government institution*, *Belgaum)*

Despite confusion, ambiguities, and concerns expressed around foreign medical research and possible side-effects arising from HIV vaccine trials, most FHSPs spoke supportively about conducting future clinical trials in Karnataka. From their experiences of working with FSWs and MSM in the context of HIV prevention programs, FHSPs appeared to be sympathetic to the struggles faced by communities in relation to their disproportionate burden of HIV. Thus, during their interviews, FHSPs reasserted the benefits that an effective HIV vaccine might offer these communities.

### Willingness to be involved in future HIV vaccine trials

Over three-quarters of the FHSP participants expressed their willingness to play a role in a hypothetical HIV vaccine trial involving FSW and MSM communities—either as a participant or in an administrative capacity. Many FHSPs felt that the relationships that they developed through on-going, close interactions with FSWs and MSM placed them in an ideal position to take part in vaccine trials and rollout programs.

*I would like to participate for the sake of the women that I have been working with so far*. *I have been working for the last six or seven years*. *I am satisfied that I have worked with these women for a noble cause*. *I feel proud thinking that I have influenced many women so as to change their life*. *I have helped many women to have a good life*. *And I am even ready to offer myself in case any additional responsibilities are given to me*. *(ORW*, *NGO*, *Belgaum)*

*[T]hese women have been suffering from so many issues*. *We need to support them* …. *[E]arlier I had misconceptions regarding these women; I was thinking that she is not good*, *she has no other jobs to do*, *she always does sex work*. *I [felt] irritated to look at them*. *A wrong notion was there*. *As I have been working with them for a long time*, *I can now understand each and every facet of their life*. *I also understand ways to encourage them*, *to motivate them*, *and what kind of support they need* …. *I am always ready to help them though I have personal work to do*. *I consider it as a service*. *(Program manager*, *CBO*, *Bellary)*

Many FHSPs believed that the training opportunities that could arise in the development of an HIV vaccine trial would substantially enhance their knowledge, skills, and experience, placing them in a better position to serve communities and contributing to their professional growth.

… *I have worked in this field for the last decade*. *I have worked with different target groups*. *Sometimes I feel that the role I have played so far has increased my knowledge* … *I always want to upgrade my knowledge and support the communities [better]*. *I wish to contribute something to this community and society*. *It helps me to grow professionally too*. *(Program manager*, *NGO*, *Belgaum)*

*I am not very educated… if we [participate in the clinical trial] we will also get information*. *In the future if the doctor does not come and explain [the study] then I can stand in front of people and explain and give information to our community members*. *We can say it is doing nothing wrong; there will be no problem*, *it is alright*. *That is why I would like to participate*. *(ORW*, *CBO*, *Bellary)*

Some FHSPs expressed altruistic sentiments toward being involved in future clinical trials, although a return could be expected by way of an increase in their social status and standing in society.

… *If my participation helps human beings* … *then* … *it is a noble cause which helps the whole mankind*. *Therefore*, *it gives some pleasure to me*. *So definitely I would like to participate*. *(Doctor*, *CBO*, *Belgaum)*

*I am very concerned about my community*. *If something good will happen to my community then I will definitely participate [in clinical trials research]*. *Gandhiji had struggled a lot to bring us freedom and he suffered a lot and he died*. *But we have not forgotten his name even today*. *Like that*, *whoever does something good for the society their name will always remain; there is no better gift than that* …. *(Program manager*, *CBO*, *Bangalore)*

A few FHSPs, particularly peer educators working with FSWs, expressed their willingness to be involved in vaccine trials as participants.

*We [FSWs] are being blamed by the public as we are into this profession*. *Therefore*, *if our participation [in vaccine trials] can benefit others*, *why shouldn’t we participate*? *We can feel good if we are able to help some people*. *(Peer educator*, *NGO*, *Belgaum)*

*I will take the vaccine first and then I will allow our community women to participate [in the vaccine trial]* …. *If something wrong happens* … *it should happen to me first* …. *I don’t want them to be in trouble*. *(Peer educator*, *CBO*, *Bellary)*

Some participants felt that playing a leadership role in vaccine trials was an extension of their responsibility as FHSPs; their long-term rapport with community members would enable them to maintain communication channels during clinical trials. Participants also felt that taking part in a trial—as a leader or research subject (in the case of ORWs and peer educators)—would better situate them to ensure the ethical conduct of clinical trials and the implementation of future HIV vaccine delivery program.

*Yes*, *if such research takes place*, *I will definitely participate* … *we will provide them [FSW and MSM community members] with appropriate information so that they can decide on their own*. *We will just explain to them about the benefits of their participation*. *We will also tell them about disadvantages*, *and let them decide for themselves*. *(ORW*, *CBO*, *Bellary)*

*I will be in a better position to recommend this to other people* … *I can communicate the objectives of vaccine trial* … *I have good rapport and am familiar with the communities’ interests* … *community members can trust us and respect our views*. *(ORW*, *CBO*, *Bangalore)*

*I have rich experience in handling their issues*. *I can prepare separate action plan for each and every FSW and MSM*. *The researchers may be new to the community*. *In such case if I come and help the research team*, *it will be effective*. *(Program manager*, *CBO*, *Bellary)*

In general, FHSPs expressed that communities would place considerable faith in their judgement with respect to clinical trials. This is corroborated by some of the participants in Chakrapani et al. [6], who reported that endorsement of clinical trials by CBO project managers was highly influential in their willingness to participate.

Irrespective of their professional background, most FHSPs expressed their commitment to playing a role in future HIV vaccine clinical trials. Specifically, opportunities to enhance their knowledge of clinical trials and to grow professionally, as well as altruistic motives and perceptions of responsibility and accountability to FSWs and MSM influenced FHSPs’ desire to be involved.

## Conclusion

This study contributes to the scant literature exploring FHSPs’ knowledge of and views on clinical trials and their potential willingness to play a role in the operation of future HIV vaccine trials. The perspectives presented by FHSPs in this study raise important implications for future planning of HIV vaccine trials among FSWs and MSM in India. Because of their on-going interactions with “high risk” communities in the context of HIV prevention services, FHSPs could be highly influential in clinical trial design, pre-study preparatory work, participant recruitment, trial implementation, and knowledge translation to community members throughout the life cycle of a vaccine trial. Importantly, and in contrast to previous findings [6,10], participants in our study—particularly those who self-identified with FSW and MSM communities—expressed great willingness to participate in future HIV vaccine trials, noting that their participation would be an extension of their duties as FHSPs. Given their rapport with community members, FHSPs could prove instrumental in effectively communicating the precise reasons why a trial to needs to shut down at an early stage, due to positive or adverse events. In other words, they can help to mitigate the circulation of misinformation that can feed negative media portrayals of clinical trials. From a communications perspective, FHSPs can also facilitate “two-way communication” by not only helping to understanding community concerns, needs, and experiences that emerge before, during and after trials, but they can also “clearly describe the research being proposed, related benefits and risks, and other practical implications” [41].

Although FHSPs are well-positioned to play leadership, ethical, and communicative roles, their apparent lack of familiarity with what clinical trials are, and the procedures involved in conducting them, suggests an important area of intervention needed before setting-up viable and ethical clinical trials in the region. For example, clinical trials specialists may need to work through any misunderstandings or negative impressions that persist among FHSPs with respect to the early termination of a gel microbicide trial in a neighbouring district in Karnataka before setting up any future HIV vaccine clinical trials in the region.

To address wider gaps in knowledge across the spectrum of FHSPs serving MSM and FSWs in this region of Karnataka requires a multi-level approach to training and capacity building as well as advocacy. For instance, curriculum development around clinical trials procedures, ethics, and meaningful community engagement should be integrated within existing medical and other health professional education programs. Physicians working in governmental and nongovernmental settings would greatly benefit from receiving specialized training around clinical trials, during which time the importance of HIV vaccine testing could be explained. CBO and NGO directors, staff, and other community leaders working with MSM and FSWs should receive capacity building so that they can effectively participate on community advisory boards that advocate for the protection of participants’ rights and interests during HIV vaccine testing.

As a qualitative study with a relatively small sample size, generalizations certainly cannot be made beyond the participant group. Moreover, our findings may not be reflective of the views of FHSPs working in government institutions; the majority of our research participants were working within the non-governmental sector, including CBOs. FHSPs working with governmental institutions might have different views and may be less willing to take part in an HIV vaccine trial due to their circumscribed responsibilities as government employees. Lastly, it cannot be assumed that communities of FSWs and MSM will unambiguously accept the FHSPs views on HIV vaccine trials.

## References

[1] International AIDS Vaccine Initiative (IAVI) (2014) Vulnerable Populations: A vaccine could help change the lives of those most vulnerable to HIV/AIDS. Available: http://www.iavi.org/publications/file/99-vulnerable-populations. Accessed 2014 Nov 9.

[2] International AIDS Vaccine Initiative (IAVI) (2014) A vaccine is essential to end AIDS. Available: http://www.iavi.org/publications/file/100-an-aids-vaccine-is-needed-to-help-end-the-pandemic. Accessed 2014 Nov 9.

[3] International AIDS Vaccine Initiative (IAVI) (2012) AIDS Vaccines: Exploring the Potential Cost/Benefit. Available: http://www.iavi.org/publications/file/69-aids-vaccines-exploring-the-potential-cost-benefit. Accessed 2014 Nov 19.

[4] National AIDS Control Organization (NACO) (2014) Annual Report 2013–14. Department of AIDS Control, Ministry of Health & Family Welfare, Government of India, Available: http://www.naco.gov.in/NACO/Quick_Links/Publication/Annual_Report/. Accessed 2014 Aug 15.

[5] Sahay S, Mehendale S, Sane S, Brahme R, Brown A, Charron K, et al (2005) Correlates of HIV vaccine trial participation: an Indian perspective. Vaccine 23: 1351–1358. 15661383 10.1016/j.vaccine.2004.09.032

[6] Chakrapani V, Newman PA, Singhal N, Jerajani J, Shunmugam M (2012) Willingness to participate in HIV vaccine trials among men who have sex with men in Chennai and Mumbai, India: a social ecological approach. PLoS One 7: e51080 10.1371/journal.pone.0051080 23226560 PMC3514227

[7] Suhadev M, Nyamathi AM, Swaminathan S, Suresh A, Venkatesan P (2009) Factors associated with willingness to participate in HIV vaccine trials among high-risk populations in South India. AIDS Res Hum Retroviruses 25: 217–224. 10.1089/aid.2007.0312 19239362 PMC13132257

[8] National AIDS Control Organisation (2012) HIV Sentinel Surveillance 2010–11: A Technical Brief. Department of AIDS Control, Ministry of Health and Family Welfare, Government of India, Available: http://www.naco.gov.in/NACO/Quick_Links/Publication/. Accessed 2015 Sep 10.

[9] AVAC-Global Advocacy for HIV Prevention (2014) AIDS Vaccines:There is momentum and promise in the search for an AIDS vaccine. Available: http://www.avac.org/prevention-option/aids-vaccines. Accessed: 2014 Nov 15.

[10] Newman PA, Chakrapani V, Weaver J, Shunmugam M, Rubincam C (2014) Willingness to participate in HIV vaccine trials among men who have sex with men in Chennai and Mumbai, India. Vaccine 32: 5854–5861. 10.1016/j.vaccine.2014.08.043 25173475

[11] National Institute of Medical Stastistics (2014) Clinical Trials Registry-India, Indian Council of Medical Research,. CTRI Website. Available: http://ctri.nic.in/Clinicaltrials/login.php. Accessed 2014 Dec 23.

[12] Mehendale S (2013) Acquired immune deficiency syndrome vaccine trials: Managing ethical issues before, during and after the trials. Perspect Clin Res 4: 84–88. 10.4103/2229-3485.106401 23533989 PMC3601713

[13] Excler JL, Kochhar S, Kapoor S, Das S, Bahri J, Ghosh MD, et al (2008) Preparedness for AIDS vaccine trials in India. Indian J Med Res 127: 531–538. 18765870

[14] Sharma DC (2001) Research halted at Indian centre accused of misconduct. Lancet 358: 992 11583767 10.1016/S0140-6736(01)06160-8

[15] Srinivasan S, Loff B (2006) Medical research in India. Lancet 367: 1962–1964. 16782469 10.1016/S0140-6736(06)68861-2

[16] Mattheij I, Pollock AM, Brhlikova P (2012) Do cervical cancer data justify HPV vaccination in India? Epidemiological data sources and comprehensiveness. J R Soc Med 105: 250–262. 10.1258/jrsm.2012.110343 22722970 PMC3380225

[17] Suba EJ, Raab SS, Viet/American Cervical Cancer Prevention P (2010) HPV vaccination: waiting for evidence of effectiveness. Lancet 375: 639–640. 10.1016/S0140-6736(10)60270-X 20171401

[18] Pandey V (2010) Cancer vaccine programme suspended after 4 girls die. DNA India Available: http://wwwdnaindiacom/india/report_cancer-vaccine-programme-suspended-after-4-girlsdie_1368681. Accessed 2014 Dec 2

[19] Dhar A (2010) Centre halts HPV vaccine project. The Hindu. New Delhi. Available: http://www.hindu.com/2010/04/08/stories/2010040857390100.htm. Accessed 2014 Nov 22.

[20] Srinivasan S, Nikarge S (2009) Ethical concerns in clinical trials in India: an investigation. Centre for Studies in Ethics and Rights, Mumbai, India. Available: http://www.cser.in/publications.php?page=4. Accessed 2015 Feb 27.

[21] Staff reporter (2001) Were banned drugs used on patients?,. The Hindu. Thiruvananthapuram, Available: http://www.thehindu.com/2001/07/29/stories/0229000n.htm. Accessed 2014 Dec 20.

[22] McNeilly PJ (2002) OHRP Determination Letter to Johns Hopkins University. Available: http://wwwhhsgov/ohrp/detrm_letrs/YR02/jun02cpdf. Accessed 2014 Dec 14.

[23] Bagla P, Marshall E (2001) Hopkins Reviews Investment In Indian Cancer Drug Trial. Science 293: 1024 11498549 10.1126/science.293.5532.1024

[24] Sarojini N, Anjali S, Ashalata S (2010) Preliminary findings of visit to Bhadrachalam: HPV Vaccine 'demonstration project' site in Andhra Pradesh. New Delhi: Sama Women's Health.

[25] McClarty LM, Lorway RR, Ramanaik S, Wylie J, Becker ML (2015) Factors influencing frontline health service providers' likelihood to recommend a future, preventive HIV vaccine to key populations in Karnataka, south India. Vaccine 33: 656–663. 10.1016/j.vaccine.2014.12.009 25528520

[26] Saxena P, Saxena R (2014) Clinical trials: changing regulations in India. Indian J Community Med 39: 197–202. 10.4103/0970-0218.143018 25364141 PMC4215498

[27] Sirohi B, Gupta S, Raghunadharao D, Shrikhande SV (2014) Clinical trials in India: At uncertain crossroads? Indian J Med Paediatr Oncol 35: 133–135. 10.4103/0971-5851.138947 25197173 PMC4152628

[28] Pandey A, Aggarwal A, Maulik M, Gupta J, Juneja A (2013) Challenges in administering a clinical trials registry: Lessons from the clinical trials registry-India. Pharmaceutical medicine 27(2): 83–93.

[29] Avahan (2008) The India AIDS Initiative, The business of HIV prevention at scale, Bill & Melinda Gates Foundation, New Delhi, India. Available: www.gatesfoundation.org/avahan. Accessed 2014 Dec 19.

[30] Allman D, Ditmore MH, Kaplan K (2014) Improving ethical and participatory practice for marginalized populations in biomedical HIV prevention trials: lessons from Thailand. PLoS One 9: e100058 10.1371/journal.pone.0100058 24949864 PMC4064984

[31] International Council of AIDS Service Organizations (ICASO) (2006) Community Involvement in HIV accine Research: Making it Work. Available: http://www.icaso.org/files/community-involvement-in-hiv-vaccine-research-making-it-work. Accessed 2014 Nov 20.

[32] Karnataka Health Promotion Trust (KHPT) (2015) Available: http://www.khpt.org/. Accessed 2014 Dec 2.

[33] Karnataka State AIDS Prevention Society (KSAPS) (2015) Directorate of Health and Family Welfare Services. Government of Karnataka. Available: http://www.ksaps.gov.in/Index.htm. Accessed 2014 Dec 2.

[34] QSR (2011) NVivo qualitative data analysis software; QSR International Pty Ltd. Version 9, 2011.

[35] Guest G, MacQueen KM, Namey EE (2012) Applied thematic analysis: Thousand Oaks, California, Sage Publications.

[36] Ritchie J, Spencer L (1994) Qualitative data analysis for applied policy research In: Bryman A, Rurgess RG, editors. Analysing Qualitative Data. London and New York: Routledge pp. 173–194.

[37] Van Damme L, Govinden R, Mirembe FM, Guedou F, Solomon S, Becker ML, et al (2008) Lack of effectiveness of cellulose sulfate gel for the prevention of vaginal HIV transmission. N Engl J Med 359: 463–472. 10.1056/NEJMoa0707957 18669425

[38] Emanuel EJ, Wendler D, Killen J, Grady C (2004) What makes clinical research in developing countries ethical? The benchmarks of ethical research. J Infect Dis 189: 930–937. 14976611 10.1086/381709

[39] Chakrapani V, Newman PA, Singhal N, Nelson R, Shunmugam M (2013) "If It's Not Working, Why Would They Be Testing It?": mental models of HIV vaccine trials and preventive misconception among men who have sex with men in India. BMC Public Health 13: 731 10.1186/1471-2458-13-731 23919283 PMC3750469

[40] McClarty LM (2013) Potential barriers and facilitators to future HIV vaccine acceptability and uptake among marginalised communities in Karnataka, south India: Perspectives of frontline health service providers Winnipeg, Canada: University of Manitoba.

[41] UNAIDS WHO (2007) Ethical considerations in biomedical HIV prevention trials. UNAIDS, Geneva 27, Switzerland.

